# Adaptive response to long-term high temperatures during the reproductive development in *Arabidopsis thaliana*

**DOI:** 10.1007/s00425-026-05108-6

**Published:** 2026-08-01

**Authors:** Juan Francisco Sánchez López, Marie Štefková, Oussama Guennich, Fen Yang, Ales Pecinka, Hélène S. Robert

**Affiliations:** 1 Faculty of Science, National Centre for Biomolecular Research, Masaryk University, 625 00 Brno, Czech Republic; 2 Plant Biotech for Stress Resilience, Genomics and Proteomics for Plant Systems, CEITEC MU-Central European Institute of Technology, Masaryk University, 625 00 Brno, Czech Republic; 3 Institute of Experimental Botany of the Czech Academy of Sciences, Centre of Plant Structural and Functional Genomics, 779 00 Olomouc, Czech Republic; 4 Present Address: State Key Laboratory for Crop Stress Resistance and High-Efficiency Production and College of Life Sciences, Northwest A and F University, Yangling, 712100 Shaanxi China

**Keywords:** Arabidopsis, Flowering, *hot1-*3, Long-term High temperature, Reproductive development

## Abstract

**Main conclusion:**

**Flowering Arabidopsis plants adapt to long-term high temperature by shortening the flowering period and reducing their fertility. The study also demonstrated that the commonly used** ***hot1-3*** **mutant is tetraploid.**

**Abstract:**

Increasing global temperatures and the rising frequency of heat waves pose a significant threat to plant reproduction. The reproductive phase is particularly sensitive to heat stress, yet the underlying mechanisms regulating thermotolerance during this stage remain insufficiently understood, despite significant advances in its understanding during vegetative growth. Heat stress responses are largely controlled by heat shock factors (HSFs) and their downstream targets, including heat shock proteins (HSPs). Among these, HSP101 is essential for acquired thermotolerance and recovery from stress, while HEAT SHOCK BINDING PROTEIN (HSBP) acts as a negative regulator of HSF activity, modulating the heat shock response. Here, we investigated the impact of elevated temperature regimes on the reproductive development of *Arabidopsis thaliana*, with a particular focus on pollen development and fertility. Our results show that heat stress negatively affects pollen development in a dose-dependent manner, leading to reduced reproductive success. We confirmed the critical role of HSP101 in reproductive thermotolerance using the *hot1-3* mutant, deficient in HSP101. Furthermore, we provide evidence that the *hot1-3* mutant is tetraploid. The origin of this event is unknown, but it is tempting to speculate that disruption of heat stress responses and interference with meiotic processes may lead to whole genome duplication. Overall, this study provides new insights into the regulation of plant reproductive development under heat stress and highlights the importance of HSP101 in maintaining fertility. These findings contribute to a better understanding of plant responses to rising temperatures and may inform strategies to enhance crop resilience under climate change.

**Supplementary Information:**

The online version contains supplementary material available at 10.1007/s00425-026-05108-6.

## Introduction

In recent decades, average global temperatures have increased due to climate change, negatively affecting ecosystems and causing losses amounting to millions of dollars in decreased food and feed production. Climate change not only results in a steady rise in global temperatures, but also leads to an increase in the frequency of extreme meteorological events, having a dramatic and adverse impact on crop production. The production of staple crops, such as rice, wheat, and soybean, is estimated to decrease significantly in the upcoming decades due to heat-induced sterility (Khan et al. 2023). High temperatures alter cellular homeostasis in plants, triggering various tolerances. These include the expression of molecular chaperones, detoxifying enzymes, such as ascorbate peroxidases, and the production of antioxidants. As sessile organisms, plants cannot escape adverse environmental conditions, leading to complex developmental responses to stresses, including heat. These responses interconnect different hormonal routes, including abscisic acid (Suzuki et al. 2016), cytokinins (Yang et al. 2016; Prerostova et al. 2020), auxin (Ai et al. 2023), ethylene (Jegadeesan et al. 2018), and brassinosteroids (Luo et al. 2022). The hormonal pathways are linked with the expression of molecular chaperones (Shi et al. 1998; Hahn et al. 2011), transcription factors (Nover et al. 2001; Koini et al. 2009; Song et al. 2018; Mizoi et al. 2019), activation of the Unfolded Protein Response (UPR), secondary metabolism and cellular responses (Avin‐Wittenberg 2018). However, in severe cases, these adaptive responses may be insufficient to counteract the damage caused by stress conditions. This can result in damaged organs, reduced fertility (Sage et al. 2015; Zhang et al. 2018), and, occasionally, plant death (Vacca et al. 2004; Wahid et al. 2007). Most studies have focused on the vegetative stage of plant development, but the reproductive phase is considered the most sensitive to heat stress (Young et al. 2004; Barnabás et al. 2008; Zinn et al. 2010; Deng et al. 2016; RECROP 2025). High temperatures can impair the development of female and male gametophytes, leading to heat-induced sterility (Endo et al. 2009; Zhang et al. 2018; Khaitova et al. 2024) and reducing fertilization of ovules (Snider et al. 2011; Sage et al. 2015), causing smaller fruits after fertilization (Chu and Chang 2020).

The HEAT SHOCK FACTORs (HSFs) are the primary regulators of the heat shock response (HSR) in plants (Scharf et al. 2012). The most notable HSF is HSFA2, which mediates the HSR (Charng et al. 2007), thermomemory (Friedrich et al. 2021), or other stress responses (Ogawa et al. 2007; Lin et al. 2018b; Zupanska et al. 2019), acting as a super activator (Chan-Schaminet et al. 2009). During the HSR, various HSFs regulate the expression of chaperones (Jacob et al. 2017), with HEAT SHOCK PROTEIN 101 (HSP101) being one of the most studied due to its role in thermotolerance (Queitsch et al. 2000; McLoughlin et al. 2019) and mediating gene expression during the stress recovery phase (Merret et al. 2017). Notably, HSP101 also mediates different aspects of plant reproduction under non-stress conditions (Fu et al. 2002; Qin et al. 2021). The *hot1-3* mutant, a knock-out mutant of the *HSP101* gene (Hong and Vierling 2001), is highly sensitive to heat and often used as a positive control in heat stress experiments (Kim et al. 2012; Wu et al. 2013; Lin et al. 2018a; Tak et al. 2022).

During the heat stress recovery phase, the HEAT SHOCK BINDING PROTEIN (HSBP) acts as a master negative regulator of the HSF transcriptional activity in eukaryotes (Satyal et al. 1998). HSBP binds to the oligomerization domain of HSFs, resulting in the dissociation of active HSF trimers to inactive monomers. The HSF monomers are then coupled to various chaperones (Fu et al. 2005; Rana et al. 2012; Huang et al. 2024). This attenuates the HSF transcriptional activity and HSR (Morimoto 1998). Under non-stress conditions, HSBP is mainly expressed in Arabidopsis siliques (Hsu et al. 2010) and regulates seed development in Arabidopsis, rice, maize and *Brassica rapa* (Fu et al. 2005; Hsu et al. 2010; Rana et al. 2012; Muthusamy et al. 2023). The Arabidopsis *hsbp-2* mutant exhibits a reduced fertilization rate accompanied by about 35% seed abortion (Hsu et al. 2010). HSBP represses HSFs by binding to the promoter of the HEI10 E3 ligase, limiting meiotic crossovers in gametes in normal and high temperatures and, thereby, influencing fertility (Kim et al. 2022). High temperatures alter meiotic crossover formation and induce the formation of unreduced gametes by interfering with cytokinesis during meiotic divisions, producing diploid and aneuploid spores (Storme and Geelen 2020).

This study aimed to provide a comprehensive analysis of the flowering phase of Arabidopsis under different high-temperature scenarios. The results offer new insights into the adaptive response to high temperatures during the plant reproductive phase. We show that high temperatures negatively affect pollen development in a dose-dependent manner and that the *hot1-3* mutant is tetraploid.

## Methods

### Seed material

We used seeds from Col-0, the GCVC pollen marker line (N67829) (Borges et al. 2012), *hsbp-2* (salk_046465) (Hsu et al. 2010), *hot1-3* (N16284) (Hong and Vierling 2001), and *hsp101* (N678620/SALK_066374C, N656804/SALK_099583C). All mutant and reporter lines are in the Col-0 accession. Primers used for genotyping *hot1-3* and *hsp101* mutant lines are provided in Supplementary Table S3.

### Growth and stress conditions

Arabidopsis seeds were sterilized using chlorine gas for five hours. Seeds were ventilated and plated on Murashige and Skoog (MS) medium. After two days of cold stratification, plates were moved to a cultivation room (Photon Systems Instruments, Czech Republic): 21/18 ºC (light/dark) with a 16-h light/8-h dark condition (LED illumination, light intensity of 150 µmol.m⁻^2^.s⁻^1^) and 50% humidity. One week after germination, plants were transferred to soil in individual pots. Plants in soil are cultivated in closed cultivation banks in phytotrons with controlled light (combination of white/red/far-red LED illumination, light intensity of 150 µmol.m⁻^2^.s⁻^1^) and temperature regimes. Arabidopsis plants remained in this condition (long-day, 21/18) until activation of the inflorescence meristem, corresponding to growth stage 5 (Boyes et al. 2001), before the elongation of the flowering stem started. At this point, plants were moved to different stress conditions or kept in this control condition (CC, 21/18) for the experiments. For Stress Condition 1 (S1, 28/18), the temperature rises from the night temperature (18 ºC) to 28 ºC in one hour in the first hour of the light period, remains stable for 14 h, and decreases to 18 ºC in the last hour of the light period. For Stress Condition 2 (S2, 28/24), the night temperature is 24 ºC and rises to 28 ºC in 2 h at the start of the light period, remains stable for 12 h, and decreases to 24 ºC in the last 2 h of the light period. For Stress Condition 3 (S3, 34/18), the temperature rises from the night temperature (18 ºC) to 34 ºC in 5 h, remains stable for 6 h, and decreases to 18 ºC in 5 h. The plants stayed in these growth conditions until they stopped flowering.

### Phenotyping of inflorescence

The length of the primary inflorescence of Col-0 plants was measured when the first flower opened in all growth conditions. Measurements were performed in intervals of five days until the end of flowering. Opened and defective flowers were counted daily during the flowering time in each growth condition. Fifteen mature siliques per plant (silique 6 to silique 21, silique 1 being from the first opened flower) from the primary inflorescence were measured to calculate the length of siliques in different conditions. A total of 20 plants were measured for all parameters in three biological replicates.

### Pollen viability and measurements

Pollen grains of at least 10 plants from all four conditions were stained with Alexander staining (Alexander 1969). Samples were taken 5 and 12 days after the beginning of flowering (DAF, Day After the start of Flowering). One flower was dipped into 15 μL of Alexander staining and vortexed vigorously. Ten microliters of the supernatant were mounted on a slide. Visualization was immediately performed using a ZEISS Axioscope.A1 microscope equipped with DIC optics and an Axiocam 506 CCD camera. The area of 150 pollen grains per plant, ten plants per treatment, was measured using Image J to determine the size of the pollen grains. To quantify pollen viability, at least 200 pollen grains were evaluated. Whole anthers were stained with Alexander staining and kept at 50 ºC for 24 h prior to visualization. The experiment was performed in at least three biological replicates.

### Pollen ploidy evaluation

Ploidy measurements were performed as described (Yang et al. 2021) with the following modifications. To determine the somatic ploidy levels, 1–2 young leaves from a 10 day-old seedling were chopped with a razor blade in 500 μL Otto I solution (0.1 M citric acid, 0.5% Tween 20 v/v). The nuclear suspension was filtered through 50 µm nylon mesh and stained with 1 mL of Otto II solution (0.4 M Na_2_HPO_4_·12H_2_O) containing 2 µg DAPI (4’,6-diamidino-2-phenylindole). The ploidy was analyzed on a Partec PAS I flow cytometer with diploid WT plants used as an external standard.

To determine ploidy levels of male gametes, the samples were prepared as described (Borges et al. 2012). The nuclei-enriched pellet was resuspended in 1 mL of LB01 buffer (15 mM TRIS, 2 mM Na_2_EDTA, 0.5 mM spermine, 80 mM KCl, 20 mM NaCl, 15 µM mercaptoethanol, 0.1% Triton X-100, pH 9) containing 2 µg DAPI. The ploidy was analyzed on FACSAria (Becton Dickinson) flow-sorter with diploid WT male gametes used as an external standard.

To visualize segregating chromosomes, mature pollen samples were prepared as described (Teng et al. 2008).

### Fertilization rate assessment

Pistils from all growth conditions were emasculated and hand-pollinated the following day with pollen of plants from the same genotype grown in 21/18 ºC conditions to achieve the maximum pollination rate possible. Siliques were dissected at two, three, and four days after pollination, and developing seeds and unfertilized ovules were counted. The experiments were performed in three biological replicates with at least 20 siliques per genotype and condition. The data from one replicate is presented. The number of counted siliques is provided in Tables 2 and 3.

### RNA extraction and cDNA synthesis

Pistils from the primary inflorescence at all growth conditions were emasculated the day before pollination. Forty unpollinated pistils were harvested at midday (at the warmest temperature), frozen on dry ice, and stored at −80 ºC. Samples were collected 5 and 12 days after flowering (DAF) began. Samples were ground in liquid nitrogen using ceramic beads and a Retsch Mixer Mill MM 400 (Qiagen). RNA was extracted following the manufacturer’s instructions using the NucleoSpin RNA Plant and Fungi Kit (Macherey–Nagel). After treatment with an RQ1 RNase-Free DNase (Promega), two micrograms of total RNA were used for cDNA synthesis using M-MLV Reverse transcriptase (Promega) following the manufacturer’s instructions.

### RT-qPCR

The FastStart Essential DNA Green Master (Roche) with SYBR Green I as the fluorescent dye was used for qPCR on a LightCycler^®^96 (Roche). Each reaction contained 5 µl FastStart Essential DNA Green Master (Roche), 2.5 µl of four times diluted cDNA sample, 500 nM gene-specific primer combinations, and PCR-grade water (Roche) to a final volume of 10 µL. The selection of the candidate reference genes, TMA7 (At1g15270) and PP2AA3 (At1g13320), for normalization was made using qBASE + (BioGazelle) and a combination of the Normfinder algorithm in R and BestKeeper Excel sheet (Vandesompele et al. 2002; Andersen et al. 2004; Taylor et al. 2019). The expression of the reference genes was not affected by the growth conditions. The primers targeting the tested transcripts are listed in Supplementary Table S3. Primers for target genes were validated by testing their efficiency and specificity. Three biological replicates were used for the experiments, each with three technical replicates. The PCR reactions consisted of a preincubation step at 95 ºC for 10 min, followed by 40 cycles of 95 °C for 10 s, 55 ºC for 10 s, and 72 ºC for 14 s. Products were checked on a 3% agarose gel. The calculation of log2-fold changes was made using the 2^DCq method according to the guidelines in Taylor et al. (2019). Statistical analysis was performed using a two-way ANOVA and post-hoc Tukey test in R Studio. A log2-fold change of 0.8 or higher was considered significant.

### Statistical analysis

To compare flowering times in the different growth conditions, a negative binomial regression test was performed, followed by ANOVA and Tukey test to make multiple pairwise comparisons. A Kaplan–Meier Survival analysis and Cox proportional hazards regression model were used to study the flowering speed, the end of flowering, and the presence of sterile flowers. One-way or two-way ANOVA and Tukey HSD tests were performed to compare the length of siliques and pollen size. To study the viability of pollen grains, number of flowers, number of ovules, number of developed seeds, non-fertilized ovules, and aborted seeds, Kruskal–Wallis, Mann–Whitney U and Dunn tests were performed, followed by a non-parametric Spearman rank correlation to establish a correlation between the number of ovules and developed seeds.

### Accession numbers

Arabidopsis AGI locus identifiers: APX2 (At3g09640), HSBP (At4g15802), HSFA1D (At1g32330), HSFA2 (At2g26150), HSFA4C (At5g45710), HSP70 (At3g12580), HSP101/HOT1 (At1g74310), PP2AA3 (At1g13320), TAA1 (At1g70560), TMA7 (At1g15270),

## Results

### Impact of long-term high temperatures on flowering rate and flower production

**Fig. 1:**
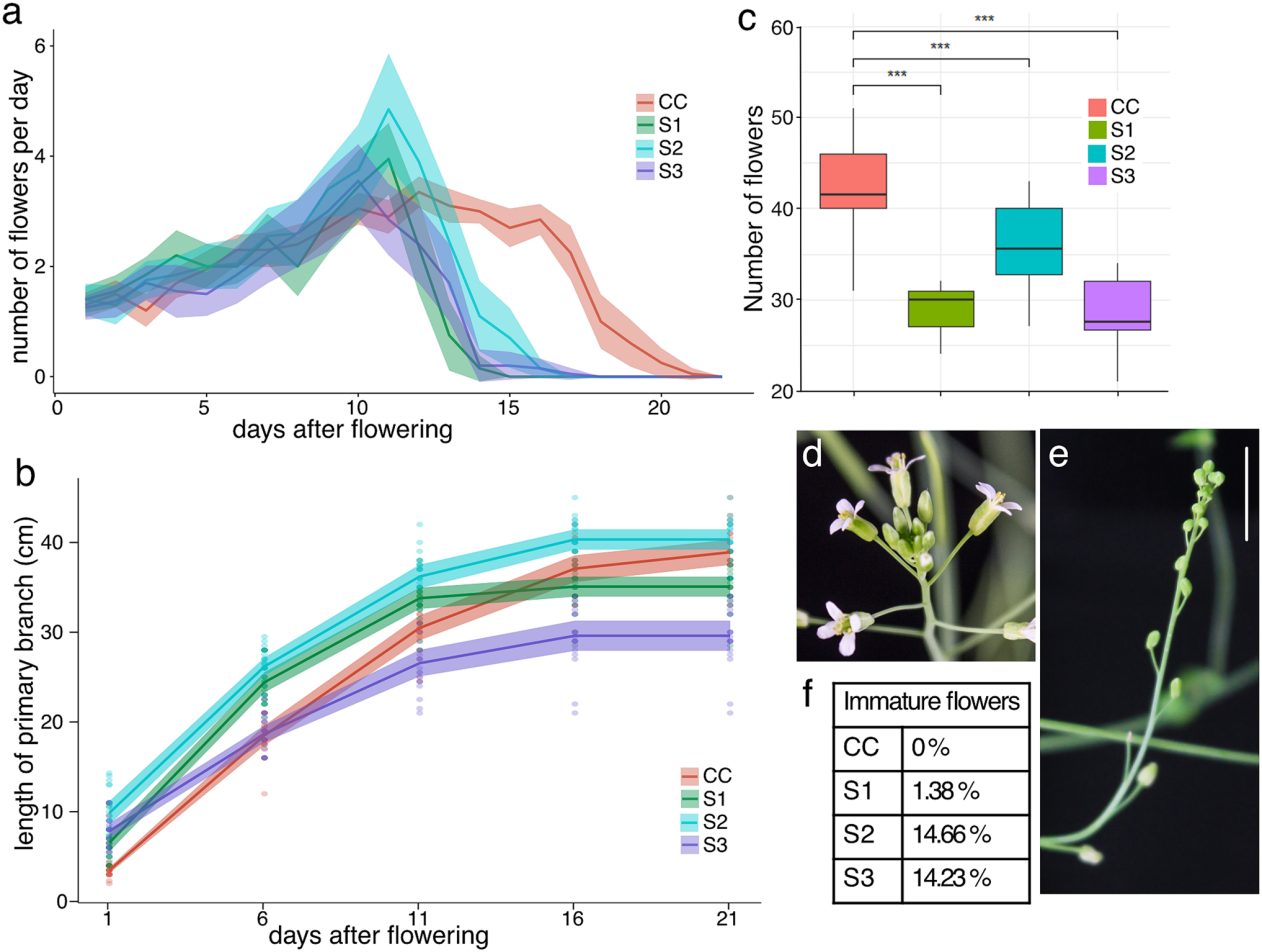
Effect of high temperatures on flower production. **a** Daily production of flowers in each growth condition on the primary inflorescence branch. Statistical analysis of the flowering rate is presented in Supplementary Table S1. **b** Length of the primary branch in cm in each growth condition. The 95% CI and mean are shown in (**a**) and (**b**). **c** Total number of flowers produced in the primary branch. Significant difference with p-value of *p* < 0.001 is indicated by ***. **d**, **e** Representative pictures of normal flowers (**d**) and immature flowers (**e**). The scale bar represents 1 cm. **f** Percentage of immature flowers produced per plant (*n* = 20). Statistical analysis using a Kaplan–Meier survival estimator and Cos proportional hazard regression is presented in Supplementary Fig. S2. *CC* control condition (21/18 °C), *S1* stress condition 1 (28/18 °C), *S2* stress condition 2 (28/24 °C), *S3* stress condition 3 (34/18 °C)

Various phenotypic changes in the flowering plants were notable when we analyze the effects of long-term high temperatures (Supplementary Figure S1). In all tested growth conditions, flowering commenced six to nine days after transferring the plants to the different stress and control conditions. The duration of flowering significantly shortened under high-temperature conditions, decreasing from approximately twenty-one days in the control condition to a range of twelve to fifteen days in all stress conditions (Fig. 1a). This reduction in flowering time affected the growth of the primary inflorescence branch, which we monitored by measuring its length at different time points (Fig. 1b). In the Control Condition (hereafter, referred to as CC), plants uniformly elongated until the end of flowering. Under Stress Condition 1 (28/18 °C, hereafter, referred to as S1) and Stress Condition 2 (28/24 °C, hereafter, referred to as S2), growth accelerated until ten days after the beginning of flowering (10 DAF), then slowed from 10 DAF to the end of flowering. Under Stress Condition 3 (34/18 °C, hereafter, referred to as S3), there was a significant reduction in the inflorescence growth rate compared to the control condition, indicating compromised inflorescence meristem activity.

Reduced flowering duration led to the production of fewer flowers (Fig. 1c). Under CC, plants produced two to four flowers daily on the primary inflorescence branch. In contrast, under stress conditions, plants produced up to eight flowers daily, with a high variability day to day (Fig. 1a). High temperatures also induced the production of flower buds that stopped their development in a dose-dependent manner, up to 14% at Stress Conditions S2 and S3 (Fig. 1d-f). This indicates that high temperatures not only reduce the primary inflorescence branch growth rate, but also reduce flower production, with a significant proportion of flowers that will not reach maturation under strong stress conditions. Inflorescence development and flower production were most affected in S2 and S3. Our statistical analysis, utilizing an exponential growth model (Supplementary Figure S2), revealed a strong correlation between inflorescence growth and flowering duration across different conditions, suggesting that high temperatures directly impact the flower meristem activity.

### Pollen grains are damaged by high-temperature stress

**Fig. 2:**
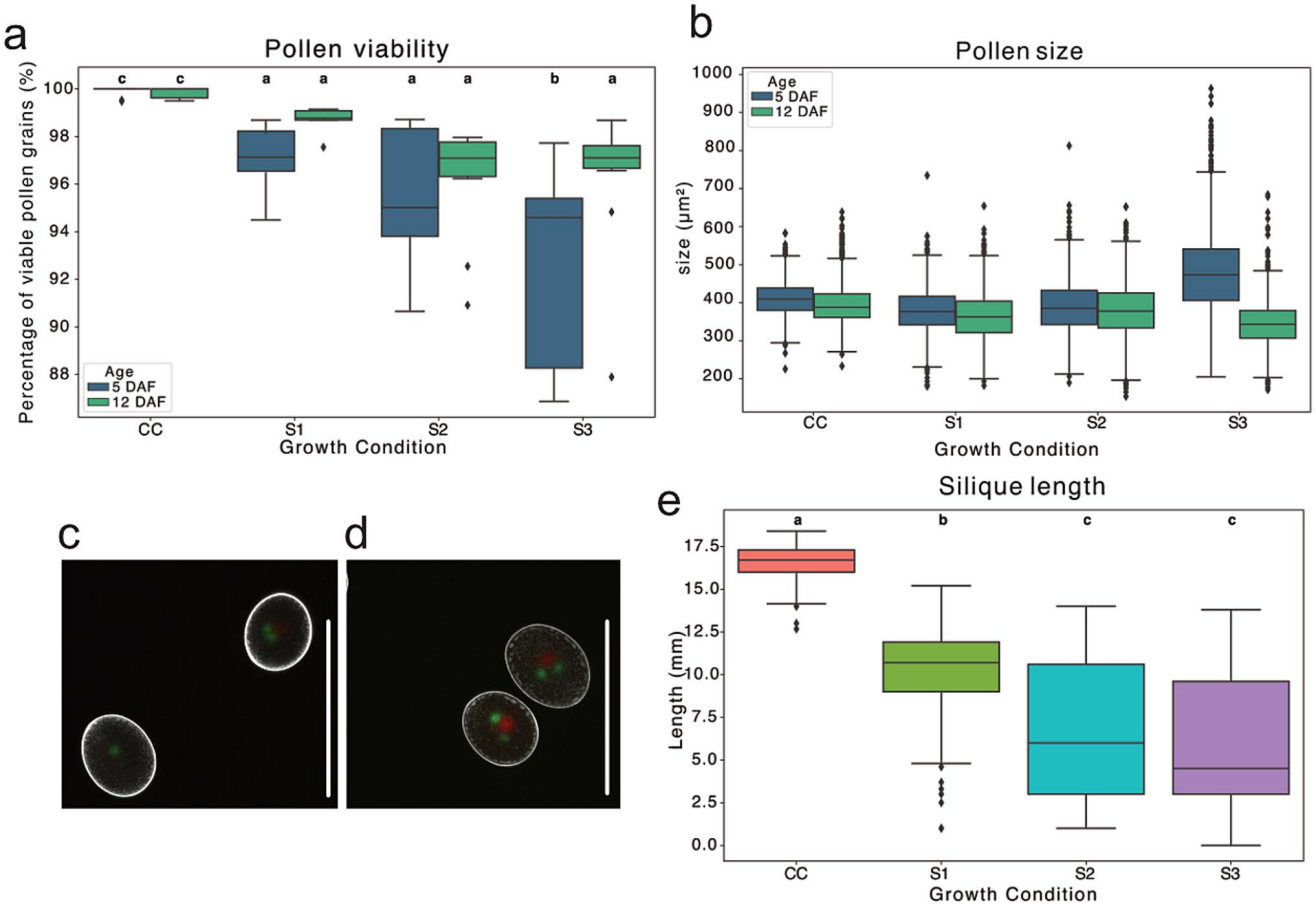
Effect of long-term high temperatures on pollen development. **a** Pollen viability assessed by counting the number of viable pollen grains from 10 plants grown under the four growth conditions (*CC* control, *S1* Stress 1, *S2* Stress 2, *S3* Stress 3) visualized by Alexander's stain at 5 and 12 Days After Flowering (DAF) (*n* = 1874—2254). Statistical analysis was performed by Kruskal–Wallis, Mann–Whitney U test and Dunn tests. Data are presented as boxplot with median value, interquartile range as box and minimum and maximum values as whiskers. Groups with the same letter are not significant. **b** Pollen size in µm^2^ from 150 grains from 10 plants grown under the four growth conditions. Statistical analysis was performed by two-way ANOVA followed by Tukey HSD tests. **c** Pollen grains from a SCVC marker plant grown at CC. The sperm cell nuclei are marked in green and the vegetative cell nucleus is marked in magenta. The scale bar is 50 µm. **d** Pollen grains from a SCVC marker plant grown at S3. The sperm cell nuclei are marked green and the vegetative cell nucleus is marked in magenta. The scale bar is 50 µm. **e** Silique length in mm from self-pollinated flowers (10 siliques) from 15 plants grown under the four growth conditions. Statistical analysis was performed by one-way ANOVA followed by Tukey HSD tests, showing significant differences between groups with *p* < 0.0001. Groups with the same letter are not significant. *CC* control condition (21/18 °C), *S1* stress condition 1 (28/18 °C), *S2* stress condition 2 (28/24 °C), *S3* stress condition 3 (34/18 °C)

To better understand how high temperatures influence seed production, we investigated the effects on pollen grains in plants grown under various high-temperature conditions. Pollen is highly sensitive to heat (Rieu et al. 2017; Raja et al. 2019). Using Alexander staining on stamens, we observed that, in CC, approximately 0.10% (*n* = 2012) of pollen grains were aborted, while in stress conditions, the percentage of aborted pollen grains in 5 DAF flowers varies from 2.95% (*n* = 2308) in S1, 4.65% (*n* = 2364) in S2, to 7.27% (*n* = 2021) in S3. Interestingly, in 12 DAF flowers, pollen grains appeared to adapt to the stress conditions with reduced abortion rates of 1.23% (*n* = 2270, S1), 3.57% (*n* = 2131, S2), and 3.85% (*n* = 2283, S3) (Table 1, Fig. 2a). This slight decrease in pollen viability at high temperatures compared to control temperature is significant at these time points.

**Table 1:** Pollen viability assessed by Alexander’s staining (*n* = 10 plants)

DAF	Condition	Aborted pollen (*n*)	% of aborted pollen	Statistical groups
5	CC	2 (2012)	0.10	a, b
12	CC	3 (2174)	0.14	a, a
5	S1	68 (2308)	2.95	a, a
12	S1	28 (2270)	1.23	b, a
5	S2	110 (2364)	4.65	a, a
12	S2	76 (2131)	3.57	a, b
5	S3	147 (2021)	7.27	a, a
12	S3	88 (2283)	3.85	b, c

Statistical analysis was performed by Kruskal–Wallis and Dunn multiple comparison tests with *p*-values adjusted by the Holm method. Non-significantly different samples were grouped by letter. The first letter for comparing the effect of time in each growth condition; and the second letter for comparing the effect of growth temperature at each time point

*DAF* days after flowering, *CC* control conditions, *S1* stress conditions 1, *S2* stress conditions 2, *S3* stress conditions 3

Remarkably, the size of pollen grains also varied between stress conditions. In CC, S1 and S2, pollen grain size was similar, averaging around 400 µm^2^. Notably, a higher number of small pollen grains (around 200 µm^2^) was only observed in stress conditions. Under S3, pollen grains in 5 DAF flowers were significantly larger, sometimes twice the size of those from the CC (Fig. 2b). These large pollen grains were not observed in 12 DAF flowers. Using the fluorescent GCVC reporter line marking distinctly the sperm cell nuclei and vegetative cell nucleus, we found no apparent changes in the number of nuclei in larger pollen grains despite their size (Fig. 2c, d).

### The fertilization rate is reduced at high temperatures

Self-pollination revealed a dose-dependent reduction in fertilization rates. The fertilization rates significantly dropped from 97.43% in CC to 79.04%, 51.31%, and 44.62% under S1, S2, and S3, respectively (Table 2). Additionally, seed abortion rates increased to 0.82% and 1.82% at S2 and S3, respectively. The correlation between the number of ovules and developed seeds (number of ovules versus number of developed seeds) weakens as temperature rises (Spearman rank correlation rho 0.95 for CC, 0.70 for S1, −0.04 for S2, −0.07 for S3), with S2 and S3 showing the least significant effects, suggesting a negative effect of the long-term high temperature on fertilization rate.

**Table 2:** Fertilization rate. developed seeds, aborted seeds, and unfertilized ovules were counted in 25 siliques per growth condition

Condition	*C*	S1	S2	S3
Silique				
*n*	25	25	25	25
Fertilized ovules				
*n*	1741	1139	627	564
%	97.43	79.04	51.31	44.62
*p*-values (compared to CC)		0.0365	< 0.0001	< 0.0001
Unfertilized ovules				
*n*	46	302	595	700
%	2.57	20.96	48.69	55.38
* p*-values (compared to CC)		< 0.0001	< 0.0001	< 0.0001
Aborted seeds				
* n*	0	0	10	23
%	0.00	0.00	0.82	1.82
* p*-values (compared to CC)		1	5.72E-02	2.30E-06
Total n	1787	1441	1222	1264
*n* ovules/silique	71.48	57.64	48.88	50.56
*p*-values (compared to CC)		< 0.0001	< 0.0001	< 0.0001
*n* dev. seeds/silique	69.64	45.56	24.68	21.64
*p*-values (compared to CC)		0.0365	< 0.0001	< 0.0001

Statistical analysis was performed by Negative Binomial regression, except for seed abortion (Kruskal–Wallis and Dunn multiple comparison tests with *p*-values adjusted by the Holm method). Aborted seeds are also counted as fertilized ovules. Developed seeds are fertilized ovules that form seeds and do not abort

*CC* control conditions, *S1* stress conditions 1, *S2* stress conditions 2, *S3* stress conditions 3

The reduced fertilization rate and increased seed abortion led to significantly fewer developed seeds per silique (Table 2). Under CC, plants averaged 69.64 developed seeds per silique, whereas this amount was reduced to 45.56, 24.68, and 21.64 under S1, S2, and S3, respectively (Table 2). This reduction in seed number also significantly affected the length of the siliques measured at flower stage 17, from 16 mm in the CC to 9.5, 7.9, and 7.4 mm in S1, S2, and S3, respectively (Fig. 2e).

### The HSR is active in temperature-stressed pistils

**Fig. 3:**
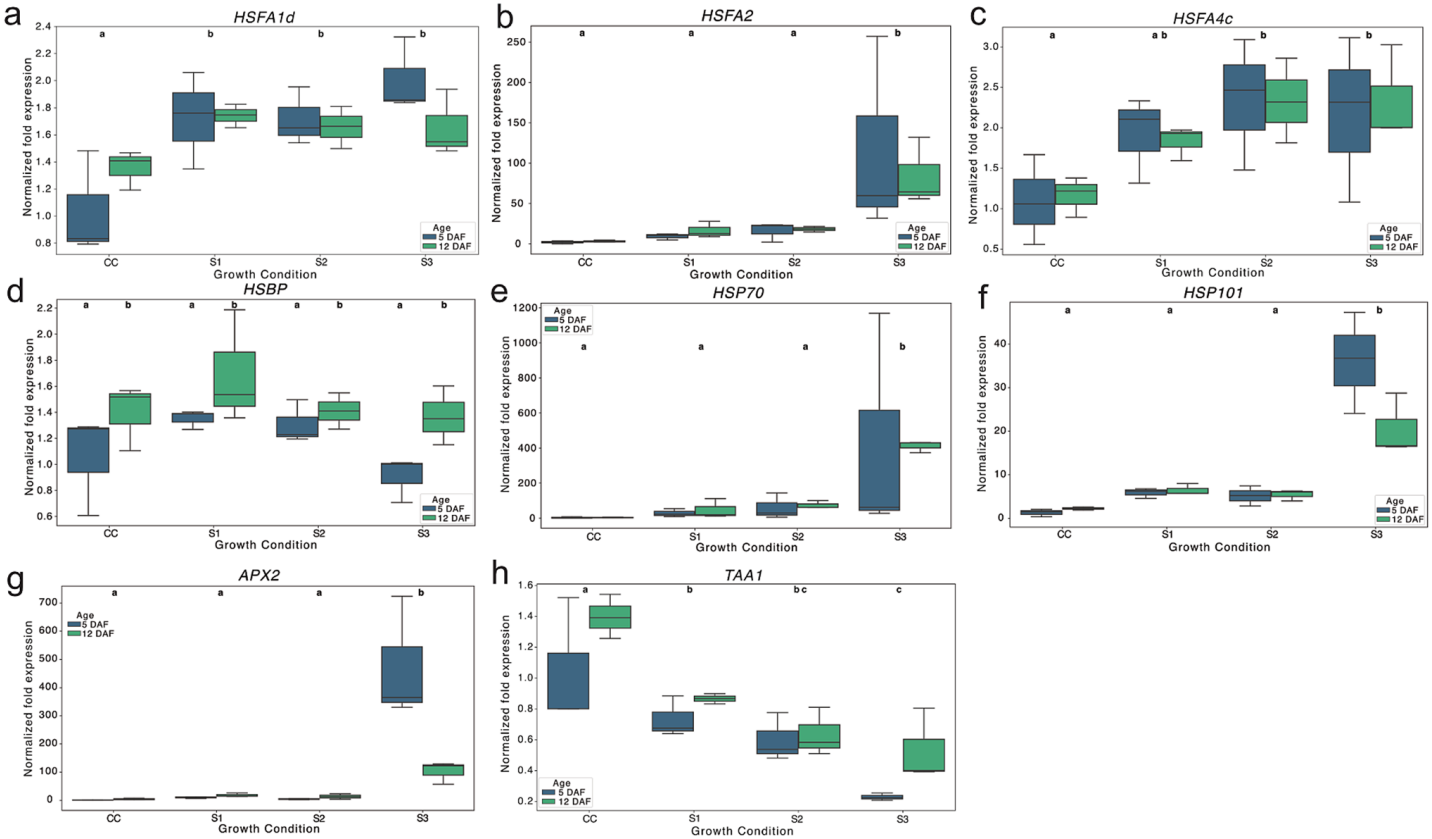
Effect of long-term high temperatures on expression of HSR-related genes in Col-0 pistils. Normalized fold expression assessed by RT-qPCR of *HSFA1d* (**a**), *HSFA2* (**b**), *HSFA4c* (**c**), *HSBP* (**d**), *HSP70* (**e**), *HSP101* (**f**), *APX2* (**g**), and *TAA1* (**h**) in pistils collected at 5 (blue) and 12 (green) DAF from plant grown under the four growth conditions (CC, S1, S2, S3). Expression levels are normalized to the expression in the 5 DAF pistils at CC. Statistical analysis was performed by two-way ANOVA followed by Tukey HSD tests, showing significant differences between groups with at least *p* < 0.05 to *p* < 0.0001. Groups with the same letter are not significant. *CC* control condition (21/18 °C), *S1* stress condition 1 (28/18 °C), *S2* stress condition 2 (28/24 °C), *S3* stress condition 3 (34/18 °C)

Heat shock and oxidative responses are crucial in managing temperature stress. To understand the role of the HSR components in response to high temperatures during fertilization, we collected unpollinated pistils at 5 and 12 DAF and analyzed the expression of the various heat-responsive genes across the different growth conditions. We focused on the expression of *HSF* genes known to be expressed in reproductive tissues (*HSFA1d*, *HSFA2*, *HSFA4c*), genes related to the HSR (*HSP70*, *HSP101*, *APX2*, *HSBP*), and auxin biosynthesis (*TAA1*) (Fig. 3). The duration of the growth at stress conditions had no impact on the expression of the tested genes, except for *HSBP* (Fig. 3d) for which a prolonged warmness led to a significantly more pronounced upregulation. The *TAA1* expression was significantly downregulated in pistils from plants grown under all stress conditions compared to plants grown in CC (Fig. 3h). *HSFA2*, *HSP70*, *HSP101*, and *APX2* were the most significantly overexpressed genes in pistils from plants grown at S3 compared to plants grown in CC and S1 and S2 (at 5 DAF, up to 6.3-, 7.0-, 5.1-, and 8.8-log_2_-fold change, respectively, Fig. 3b, e–g). To a lesser extent, *HSFA1d* and *HSFA4c* genes were also significantly upregulated in pistils from plants grown under S2 and S3 compared to plants grown in CC (at 5 DAF, 1- and 1.1- log_2_-fold change, respectively, Fig. 3a and c). *HSFA1d*, *HSFA4c* and *HSBP* were only mildly affected by the growth conditions. It is important to note that *HSF* transcripts can undergo post-transcriptional regulation, affecting their activity and stability in the cell (Cohen-Peer et al. 2010; Evrard et al. 2013; Carranco et al. 2017; Andrási et al. 2019). Therefore, even small upregulations in these transcription factors can significantly impact cellular responses, as they may already be present in an inactivated state. In addition, this gene expression analysis indicated that S3 appears to be the most stressful and that gene expression patterns under S1 and S2 were rather similar. Therefore, we decided to continue our analysis only with plants grown at S1 and S3.

### The *hot1-3 *and *hsbp-2* mutant pollen is defective

**Fig. 4:**
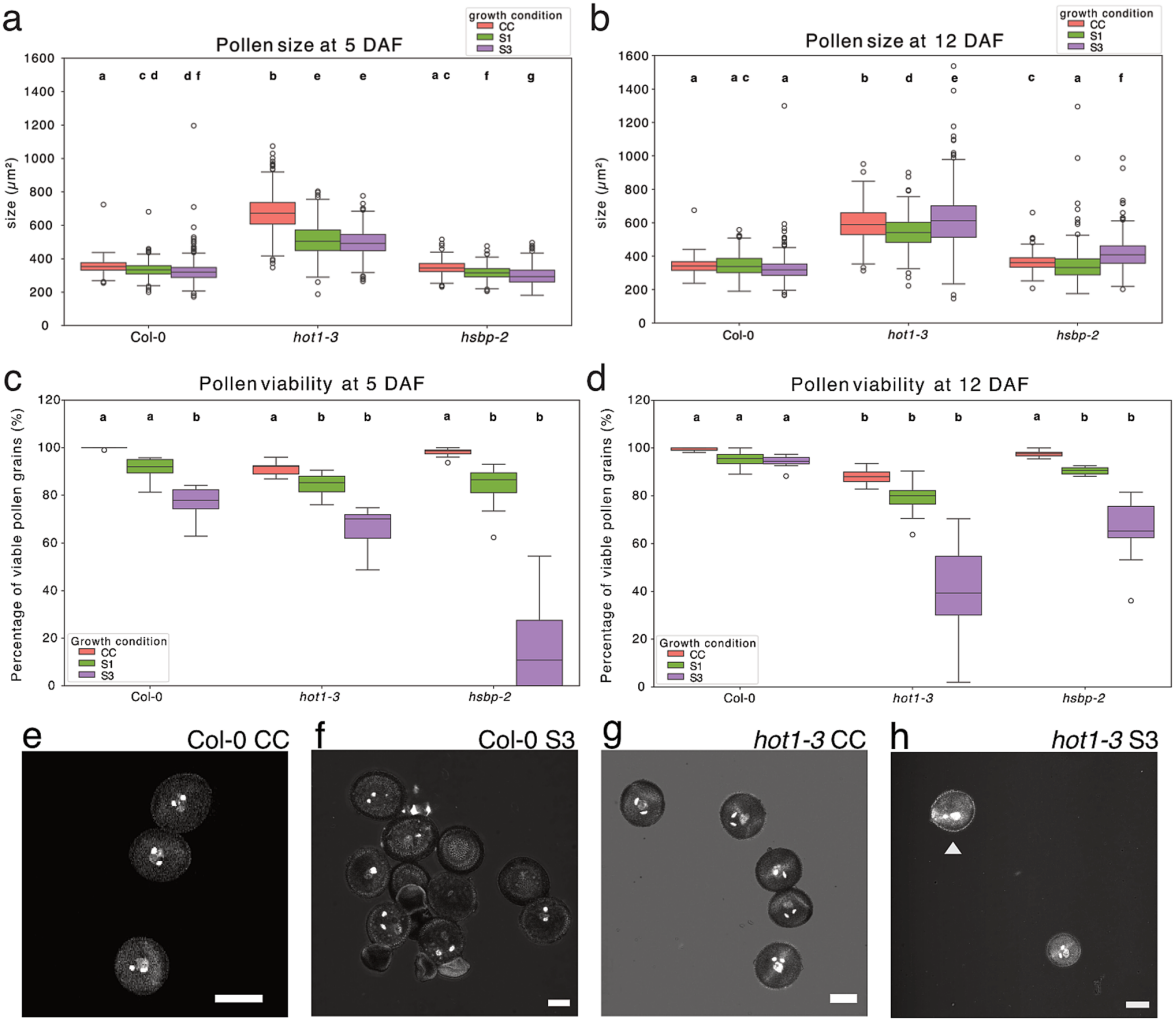
Effect of long-term high temperatures on pollen development in *hot1-3* and *hsbp-2* mutant. **a**, **b** Pollen size in µm^2^ from 500 grains from 10 plants (Col-0, *hot1-3* and *hsbp-2*) grown under the three growth conditions (*CC* control (red), *S1* Stress 1 (green), *S3* Stress 3 (purple)), assessed at 5 and 12 Days After Flowering (DAF) (*n* = 1874–2254). Statistical analysis was performed by two-way ANOVA followed by Tukey HSD tests. **c**, **d** Pollen viability assessed by counting the number of viable pollen grains from 10 plants grown under the three growth conditions (*CC* control (red), *S1* Stress 1 (green), *S3* Stress 3 (purple)) visualized by Alexander’s stain at 5 and 12 Days After Flowering (DAF). Statistical analysis was performed by Kruskal–Wallis, Mann–Whitney U test and Dunn tests. Data are presented as boxplot with median value, interquartile range as box and minimum and maximum values as whiskers. Groups with the same letter are not significant. **e**–**h** Pollen grains of Col-0 (**e**, **f**) and *hot1-3* (**g**, **h**) grown under CC (**e**, **g**) and S3 (**f**, **h**) growth conditions. Pollen nuclei were stained by DAPI. Scale bars are 20 μm. **h** Note that pollen grains of two sizes are shown (the arrowhead points to the large one). *CC* control condition (21/18 °C), *S1* stress condition 1 (28/18 °C), *S2* stress condition 2 (28/24 °C), *S3* stress condition 3 (34/18 °C)

The expression analysis indicates that S3 most severely affected the development of reproductive organs. The *hot1-3* and *hspb-2* mutants, which are impaired in HSP101 and HSBP, respectively, are known for their heat-sensitivity (Hong and Vierling 2001; Hsu et al. 2010) and reproductive issues in non-stress conditions (Fu et al. 2002; Qin et al. 2021). We phenotyped the mature pollen of both mutants under CC, mild stress (S1), and severe stress (S3). Pollen development was compromised in both mutant lines. The pollen defects and viability frequencies were similar in the *hsbp-2* mutant and the wild type in CC. Still, S3 caused complete sterility in the *hsbp-2* pollen at 5 DAF (Fig. 4c). Partial recovery was observed at 12 DAF with 70% viable *hsbp-2* pollen (Fig. 4d). On the other hand, 10% of *hot1-3* pollen grains were aborted in CC, reaching 32% at 5 DAF and 57% at 12 DAF under S3, indicating an absence of acclimation (Fig. 4c, d).

At 5 DAF, both Col-0 and *hsbp-2* pollen grains reduced their size, with an increased number of outliers for larger and smaller grains in all stress conditions (Fig. 4a). Notably, at 12 DAF, the number of outliers decreased for Col-0, but not in the *hsbp-2* mutant, which produced larger pollen grains at S3 compared to CC (Fig. 4b). The *hot1-3* mutant produced larger pollen grains, twice the size of the Col-0 ones under CC. The overall size of the *hot1-3* pollen grains was reduced at 5 DAF under both stress conditions when compared to CC (Fig. 4a). Under S3 at 12 DAF, while the average size of the *hot1-3* pollen grains was comparable to their size under CC, outliers were twice or three times the standard size (Fig. 4b).

The defects in pollen grains in *hsbp-2* translated into a decreased fertilization rate and seed production (Table 3). In the CC, 39.29% of ovules were unfertilized (*n* = 2751), and 22.10% of the developing seeds were aborted in *hsbp-2* mutant, reproducing published data (Hsu et al. 2010). Under stress conditions 1 and 3, 69.62% and 95.56% of *hsbp-*2 ovules were unfertilized, producing only 30.38% and 4.44% seeds, respectively, among which 20.86% and 58.54% aborted. The *hsbp-2* mutants exhibit severe reproductive defects under high-temperature stress. These findings underscore the importance of HSBP and the HSR pathway in maintaining reproductive development under stress conditions.

**Table 3:** Fertilization rate in Col-0 and *hsbp-2*. Developed seeds, aborted seeds, and unfertilized ovules were counted in n siliques per growth condition

Genotypes	Col-0			*hspb-2*		
Condition	CC	S1	S3	CC	S1	S3
Silique						
* n*	25	24	25	54	22	50
Ovules						
*n*	1806	1386	1096	2751	1152	1848
Ovules/siliques						
* n*	72.24	57.75	43.84	50.94	52.36	36.96
p-values (compared to CC of the same genotype)		< 0.0001	< 0.0001		1	< 0.0001
p-values (compared to Col-0 from the same conditions)				< 0.0001	0.0805	< 0.0001
Fertilized ovules						
* n*	1804	1386	1063	1670	350	82
%	99.89	100.00	96.99	60.71	30.38	4.44
Unfertilized ovules						
* n*	2	0	33	1081	802	1766
%	0.11	0.00	3.01	39.29	69.62	95.56
p-values (compared to CC of the same genotype)		1	0.0376		< 0.0001	< 0.0001
p-values (compared to Col-0 from the same conditions)				< 0.0001	< 0.0001	< 0.0001
Developed seeds						
* n*	1794	1134	674	1301	277	34
% of fertilized ovules	99.45	81.82	63.41	77.90	79.14	41.46
p-values (compared to CC of the same genotype)	< 0.0001	< 0.0001		< 0.0001	< 0.0001	< 0.0001
p-values (compared to Col-0 from the same conditions)				< 0.0001	< 0.0001	< 0.0001
ABORTED seeds						
*n*	10	252	389	369	73	48
% of fertilized ovules	0.55	18.18	36.59	22.10	20.86	58.54
p-values (compared to CC of the same genotype)	< 0.001	0.0003		< 0.0001	< 0.0001	< 0.001
p-values (compared to Col-0 from the same conditions)				< 0.0001	0.9288	< 0.0001

Statistical analysis was performed on Kruskal–Wallis and Dunn multiple comparison tests with *p*-values adjusted by the Holm method. Aborted seeds are also counted as fertilized ovules. Developed seeds are fertilized ovules that form seeds that do not abort

*CC* control conditions, *S1* stress conditions 1, *S3* stress conditions 3

### *hot1-3* is tetraploid and can develop aneuploidy

**Fig. 5:**
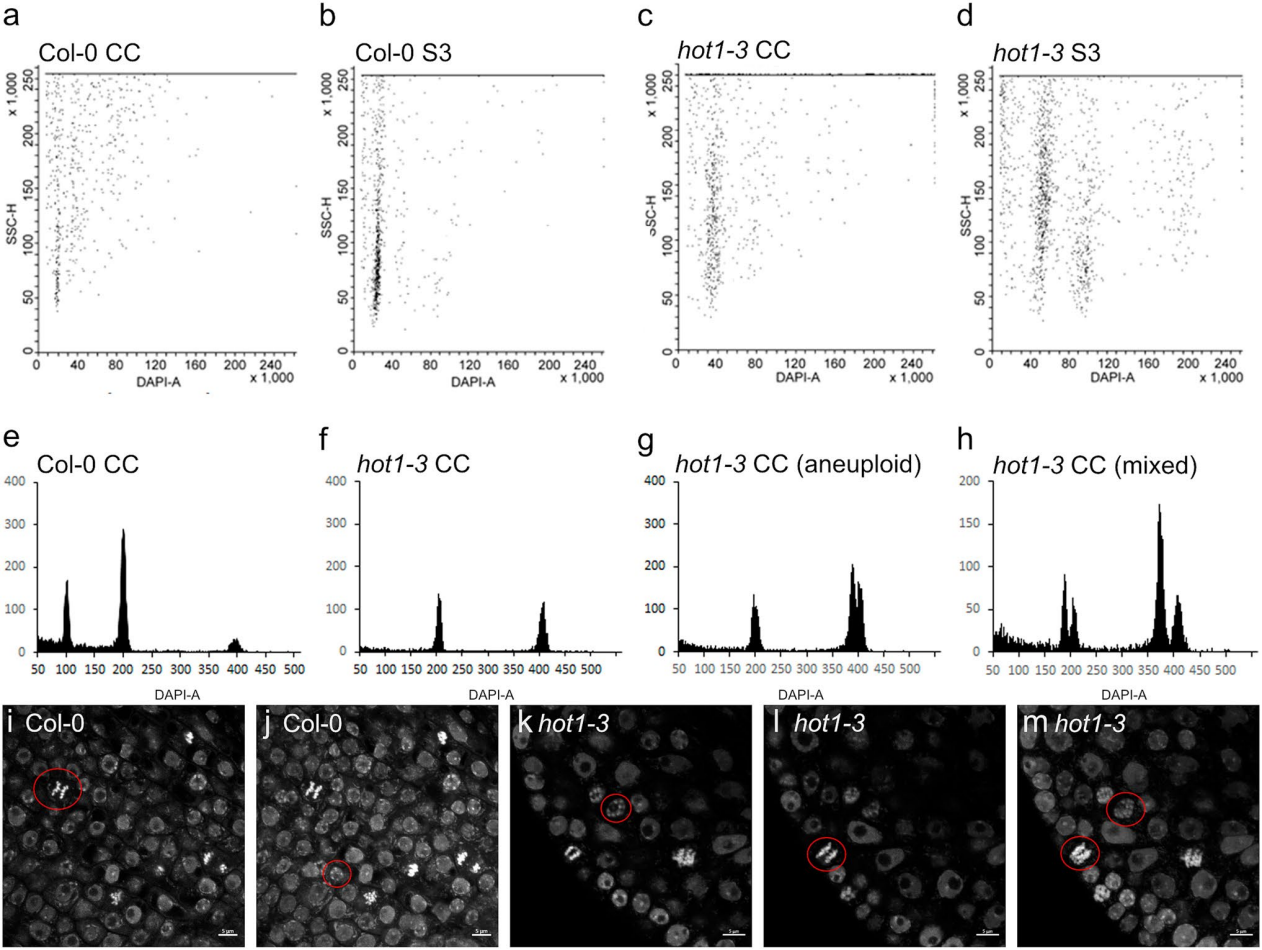
*hot1-3* is tetraploid and aneuploid. **a**-**d** Bivariate scatter plot of sperm nuclei from Col-0 (a, b) and *hot1-3* mutant (c, d) plants grown under the CC (**a**, **c**) and S3 (**b**, **d**) growth conditions. The x-axis shows relative DAPI intensity (DNA content) and the y-axis side scatter pulse height (SSC-h), indicating the running time of the nuclei through the sensor. **e**–**h** Flow cytometry histograms showing representative profiles of peaks from diploid Col-0 (**e**) and *hot1-3* mutant **f**–**h** plants grown under the CC growth condition. The first peak corresponds to 2 C, the second to 4 C and the third to 8 C nuclei (8C peak not shown in *hot1-*3). The histograms show two types of ploidy in *hot1-3* mutant: tetraploid (**f**) and aneuploid (**g**). The histogram in (**h**) displays the overlap when the material of the two plants is mixed prior analysis. **i**-**m** Representative pictures (**i**, **k**, **l**) and projection of 5–8 images (**j**, **m**) from sepals of Col-0 (**i**, **j**) and *hot1-3* (**k**-**m**) flowers. The red circles points to cells where chromosomes are visible (5 is j and k compared to 10 in k-m). Nuclei are staining by DAPI. Scale bar is 5 μm

Given the size of *hot1-3* pollen grains, we investigated whether the mutant had issues with mitotic divisions during pollen development. None of the evaluated pollen grains, stained by DAPI, had an abnormal number of nuclei, regardless of the growth condition or size of the pollen grains (Fig. 4e-h). Next, we assessed the ploidy of Col-0 and *hot1-3* sperm nuclei from CC and S3 using flow cytometry. Col-0 consistently showed a haploid DNA content cluster of the diploid parent at 20 DAPI signal units (Fig. 5a, b), regardless of the temperature regime. However, the *hot1-3* sperm nuclei displayed two clusters corresponding to haploid DNA content (40–50 DAPI signal units) and likely diploid DNA content (100 DAPI signal units) under S3 of tetraploid parents. This result indicated two unexpected findings: (i) *hot1-3* plants were tetraploid and (ii) produced unreduced microspores (Fig. 5c, d). We confirmed the tetraploidy of *hot1-3* plants in leaf samples (Fig. 5e-h, Supplementary Fig. S3). Furthermore, staining anaphase chromosomes with DAPI identified ten segregating chromosomes in Col-0 sepals, but twenty chromosomes in *hot1-3* sepals (Fig. 5k-m). Overlaying the chromatographs from leaves revealed a double peak at 200 DAPI signal units and two peaks at 350 and 400 DAPI signal units, identifying that some *hot1-3* plants are also aneuploid (Fig. 5h). Also, more dispersed appearance of the flow cytometry clusters for *hot1-3* sperm nuclei compared to wild-type ones indicates varying DNA contents within one ploidy (Fig. 5a-c), indirectly implying presence of extensive aneuploidy.

To confirm whether the ploidy change was due to the inactivation of HSP101, we assessed the ploidy from the original *hot1-3* seeds obtained from NASC (N16284) and two other *hsp101* mutant alleles (SALK_066374C and SALK_099583C) (Supplementary Fig. S4). The original *hot1-3* mutant plants were tetraploid, but the two other *hsp101* mutant lines were diploid (Supplementary Fig. S3), suggesting that the *hot1-3* became tetraploid at some point during the selection and characterisation of the mutant allele (Mathur et al. 1998; Hong and Vierling 2001).

## Discussion

In recent years, the global climate crisis has significantly reduced crop production (Sidhu 2023), highlighting the need to understand plant adaptive strategies in response to high temperatures. To address this, we studied different high-temperature scenarios using *Arabidopsis thaliana* (this work) and *Brassica napus* (Mácová et al. 2022) as model plants. Previous research primarily focused on plant reactions to short heat stress, which is less representative of natural conditions. In our study, we designed different nature-like scenarios that allow plants to grow and adapt to different heat stress conditions, providing comprehensive insights into plant thermo-adaptation to high-temperature environments.

Our study found that flowering in Arabidopsis at 34 ºC results in a reduced number of fertile flowers and decreased fertility. Previous studies have linked this reduction in crop production to heat-induced pollen sterility (Endo et al. 2009; Begcy et al. 2019). While high temperatures did affect pollen development in our growth setup, it adapted to long-term heat stress, likely due to cooler night temperatures. Unlike our approach, most Arabidopsis studies use intense heat shocks (Lei et al. 2020; Rutley et al. 2021) or constant high temperatures over long periods (Yoshida et al. 2008; Hedhly et al. 2020), conditions that are uncommon in nature and cause severe plant damages. High temperatures also affect the female gametophyte in other plant species (Wang et al. 2021; Shi et al. 2022; Jedličková et al. 2023), but this process is not understood in *Arabidopsis thaliana*. Consequently, damage to both gametophytes may result in reduced pollination and fertilization rates, significantly decreasing seed production (Jiang et al. 2019).

Expression profiling showed that 34 ºC severely affects plant metabolism. Stress-related genes, such as *HSP101*, *HSP70*, and *APX2*, were extremely upregulated in pistils of plants grown under stress conditions, while transcription factors (*HSFA2*, *HSFA1d*, or *HSFA4c*) regulating HSR were upregulated to a lesser extent. Chaperones, such as HSP101, are key to thermo-adaptation (Gurley 2000; Queitsch et al. 2000; Hong and Vierling 2001; Nieto-Sotelo et al. 2002; Wu et al. 2013). Adverse environments induce Reactive Oxygen Species (ROS) production, such as H_2_O_2_, which must be detoxified, notably by peroxidases, for example during abiotic stresses (Chang et al. 2009), as well as during developmental processes (Ye and Tran 2000; Jacobowitz et al. 2019).

The Arabidopsis *hot1-3* mutant, characterized by a T-DNA insertion in the second exon of the *HSP101* gene (Hong and Vierling 2001), displays severe defects during male gametophyte development when grown under high temperature conditions. This underscores the critical role of HSP101 in protecting gametes during thermal stress. Optimal temperatures are essential for meiotic crossovers (Lloyd et al. 2018) and chromosome segregation (Lei et al. 2020; Khaitova et al. 2024). Our study revealed that the *hot1-3* mutant is tetraploid. This line was isolated from a T-DNA insertion population generated from *Agrobacterium*-mediated transformation of *Arabidopsis* cell suspensions (Mathur et al. 1998). Selected calli were regenerated using classical tissue culture techniques. The resulting plants may exhibit alteration in ploidy and chromosome number or structure due to genome instability inherent to the tissue culture environment (Fras and Maluszynska 2003; Bednarek and Orłowska 2020). Although it is unclear when the polyploidization occurred, it may likely be during its early generation as it is already present in the material donated to the stock centre by Suk-Whan Hong (Hong and Vierling 2001). Interestingly, our data showed that *hot1-3* developed aneuploidy under high temperatures. However, because *hot1-*3 was the only line evaluated for this phenotype, it remains unclear whether this susceptibility is directly caused by the loss of HSP101 function or if it stems from the specific genomic background of this line. Heat stress is known to induce unreduced microspores (Storme and Geelen 2020), as observed in Arabidopsis Col-0 plants grown at 35/22 ºC (Nguyen et al. 2019). However, this effect was not seen under our Stress Condition 3, which could be attributed to the different temperature fluctuations between day and night (13 °C in Nguyen et al. (2019) versus 18 °C in our study).

HSFs coordinate both the HSR and plant development. HSBP, a negative regulator of HSF transcriptional activity, attenuates the HSR when temperatures decrease (Hsu et al. 2010). Using the *hsbp-2* mutant, which cannot efficiently attenuate the HSR, we examined how Arabidopsis seeds develop under our stress conditions. HSBP regulates seed development (Hsu et al. 2010) and meiotic crossover during ovule development (Kim et al. 2022). Our findings show that *hsbp-2* pollen development is susceptible to high temperatures. The analysis of a hypersensitive mutant to temperature stress (*hot1-3*) and a hyperreactive mutant (*hsbp-2*) provided insights into the reproductive phase under temperature stress.

## Supplementary Information

Below is the link to the electronic supplementary material.

Supplementary file1 (PDF 101 kb)

Supplementary file2 (PDF 4620 kb)

## Data Availability

Source data are provided in the Zenodo repository (10.5281/zenodo.19607439).

## References

[CR1] Ai H, Bellstaedt J, Bartusch KS et al (2023) Auxin‐dependent regulation of cell division rates governs root thermomorphogenesis. EMBO J 42:EMBJ2022111926. 10.15252/embj.2022111926 10.15252/embj.2022111926 PMC10233379 37071525

[CR2] Alexander MP (1969) Differential staining of aborted and nonaborted pollen. Stain Technol 44:117–122. 10.3109/10520296909063335 4181665 10.3109/10520296909063335

[CR3] Andersen CL, Jensen JL, Ørntoft TF (2004) Normalization of real-time quantitative reverse transcription-PCR data: a model-based variance estimation approach to identify genes suited for normalization, applied to bladder and colon cancer data sets. Cancer Res 64:5245–5250. 10.1158/0008-5472.can-04-0496 15289330 10.1158/0008-5472.CAN-04-0496

[CR4] Andrási N, Rigó G, Zsigmond L et al (2019) The mitogen-activated protein kinase 4-phosphorylated heat shock factor A4A regulates responses to combined salt and heat stresses. J Exp Bot 70:4903–4918. 10.1093/jxb/erz217 31086987 10.1093/jxb/erz217 PMC6760271

[CR5] Avin-Wittenberg T (2018) Autophagy and its role in plant abiotic stress management. Plant Cell Environ 42:1045–1053. 10.1111/pce.13404 29998609 10.1111/pce.13404

[CR6] Barnabás B, Jäger K, Fehér A (2008) The effect of drought and heat stress on reproductive processes in cereals. Plant Cell Environ 31:11–38. 10.1111/j.1365-3040.2007.01727.x 17971069 10.1111/j.1365-3040.2007.01727.x

[CR7] Bednarek PT, Orłowska R (2020) Plant tissue culture environment as a switch-key of (epi)genetic changes. Plant Cell Tissue Organ Cult (PCTOC) 140:245–257. 10.1007/s11240-019-01724-1

[CR8] Begcy K, Nosenko T, Zhou L-Z et al (2019) Male sterility in maize after transient heat stress during the tetrad stage of pollen development. Plant Physiol 181:683–700. 10.1104/pp.19.00707 31378720 10.1104/pp.19.00707 PMC6776839

[CR9] Borges F, Gardner R, Lopes T et al (2012) FACS-based purification of *Arabidopsis* microspores, sperm cells and vegetative nuclei. Plant Methods 8:44. 10.1186/1746-4811-8-44 23075219 10.1186/1746-4811-8-44 PMC3502443

[CR10] Boyes DC, Zayed AM, Ascenzi R et al (2001) Growth stage-based phenotypic analysis of *Arabidopsis*. Plant Cell 13:1499–1510. 10.1105/tpc.010011 11449047 10.1105/TPC.010011 PMC139543

[CR11] Carranco R, Prieto-Dapena P, Almoguera C, Jordano J (2017) SUMO-dependent synergism involving heat shock transcription factors with functions linked to seed longevity and desiccation tolerance. Front Plant Sci 8:974. 10.3389/fpls.2017.00974 28659940 10.3389/fpls.2017.00974 PMC5468958

[CR12] Chang CCC, Ślesak I, Jordá L et al (2009) *Arabidopsis* chloroplastic glutathione peroxidases play a role in cross talk between photooxidative stress and immune responses. Plant Physiol 150:670–683. 10.1104/pp.109.135566 19363092 10.1104/pp.109.135566 PMC2689974

[CR13] Chan-Schaminet KY, Baniwal SK, Bublak D et al (2009) Specific interaction between tomato HsfA1 and HsfA2 creates hetero-oligomeric superactivator complexes for synergistic activation of heat stress gene expression. J Biol Chem 284:20848–20857. 10.1074/jbc.m109.007336 19491106 10.1074/jbc.M109.007336 PMC2742850

[CR14] Charng Y, Liu H, Liu N et al (2007) A heat-inducible transcription factor, Hsf A2, is required for extension of acquired thermotolerance in *Arabidopsis*. Plant Physiol 143:251–262. 10.1104/pp.106.091322 17085506 10.1104/pp.106.091322 PMC1761974

[CR87] Chu Y, Chang J (2020) High temperature suppresses fruit/seed set and weight and cladode regreening in red-fleshed ‘Da Hong’ Pitaya (Hylocereus polyrhizus) under controlled conditions. HortScience 55(8):1259–1264. 10.21273/HORTSCI15018-20

[CR15] Cohen-Peer R, Schuster S, Meiri D et al (2010) Sumoylation of *Arabidopsis* heat shock factor A2 (HsfA2) modifies its activity during acquired thermotholerance. Plant Mol Biol 74:33–45. 10.1007/s11103-010-9652-1 20521085 10.1007/s11103-010-9652-1

[CR16] Deng Y, Srivastava R, Quilichini TD et al (2016) IRE1, a component of the unfolded protein response signaling pathway, protects pollen development in *Arabidopsis* from heat stress. Plant J 88:193–204. 10.1111/tpj.13239 27304577 10.1111/tpj.13239

[CR17] Endo M, Tsuchiya T, Hamada K et al (2009) High temperatures cause male sterility in rice plants with transcriptional alterations during pollen development. Plant Cell Physiol 50:1911–1922. 10.1093/pcp/pcp135 19808807 10.1093/pcp/pcp135

[CR18] Evrard A, Kumar M, Lecourieux D et al (2013) Regulation of the heat stress response in *Arabidopsis* by MPK6-targeted phosphorylation of the heat stress factor HsfA2. PeerJ 1:e59. 10.7717/peerj.59 23638397 10.7717/peerj.59 PMC3628891

[CR19] Fras A, Maluszynska J (2003) Regeneration of diploid and tetraploid plants of *Arabidopsis thaliana* via callus. Acta Biol Cracov Ser Bot 45:145–152

[CR20] Friedrich T, Oberkofler V, Trindade I et al (2021) Heteromeric HSFA2/HSFA3 complexes drive transcriptional memory after heat stress in *Arabidopsis*. Nat Commun 12:3426. 10.1038/s41467-021-23786-6 34103516 10.1038/s41467-021-23786-6 PMC8187452

[CR21] Fu S, Meeley R, Scanlon MJ (2002) Empty pericarp2 encodes a negative regulator of the heat shock response and is required for maize embryogenesis. Plant Cell 14:3119–3132. 10.1105/tpc.006726 12468731 10.1105/tpc.006726 PMC151206

[CR22] Fu S, Rogowsky P, Nover L, Scanlon MJ (2005) The maize heat shock factor-binding protein paralogs EMP2 and HSBP2 interact non-redundantly with specific heat shock factors. Planta 224:42–52. 10.1007/s00425-005-0191-y 16331466 10.1007/s00425-005-0191-y

[CR23] Gurley WB (2000) HSP101: a key component for the acquisition of thermotolerance in plants. Plant Cell 12:457. 10.2307/3871060 10760235 10.1105/tpc.12.4.457 PMC526003

[CR24] Hahn A, Bublak D, Schleiff E, Scharf K-D (2011) Crosstalk between Hsp90 and Hsp70 chaperones and heat stress transcription factors in tomato. Plant Cell 23:741–755. 10.1105/tpc.110.076018 21307284 10.1105/tpc.110.076018 PMC3077788

[CR25] Hedhly A, Nestorova A, Herrmann A, Grossniklaus U (2020) Acute heat stress during stamen development affects both the germline and sporophytic lineages in *Arabidopsis thaliana* (L.) Heynh. Environ Exp Bot 173:103992. 10.1016/j.envexpbot.2020.103992

[CR26] Hong SW, Vierling E (2001) Hsp101 is necessary for heat tolerance but dispensable for development and germination in the absence of stress. Plant J Cell Mol Biol 27:25–35 10.1046/j.1365-313x.2001.01066.x 11489180

[CR27] Hsu S-F, Lai H-C, Jinn T-L (2010) Cytosol-localized heat shock factor-binding protein, AtHSBP, functions as a negative regulator of heat shock response by translocation to the nucleus and is required for seed development in *Arabidopsis*. Plant Physiol 153:773–784. 10.1104/pp.109.151225 20388662 10.1104/pp.109.151225 PMC2879799

[CR28] Huang Y-C, Liu C-C, Li Y-J et al (2024) Multifaceted roles of *Arabidopsis* heat shock factor binding protein in plant growth, development, and heat shock response. Environ Exp Bot 226:105878. 10.1016/j.envexpbot.2024.105878

[CR29] Jacob P, Hirt H, Bendahmane A (2017) The heat-shock protein/chaperone network and multiple stress resistance. Plant Biotechnol J 15:405–414. 10.1111/pbi.12659 27860233 10.1111/pbi.12659 PMC5362687

[CR30] Jacobowitz JR, Doyle WC, Weng JK (2019) PRX9 and PRX40 are extensin peroxidases essential for maintaining tapetum and microspore cell wall integrity during *Arabidopsis* anther development. Plant Cell 31:848–861. 10.1105/tpc.18.00907 30886127 10.1105/tpc.18.00907 PMC6501601

[CR31] Jedličková V, Hejret V, Demko M et al (2023) Transcriptome analysis of thermomorphogenesis in ovules and during early seed development in *Brassica napus*. BMC Genomics 24:236. 10.1186/s12864-023-09316-2 37142980 10.1186/s12864-023-09316-2 PMC10158150

[CR32] Jegadeesan S, Beery A, Altahan L et al (2018) Ethylene production and signaling in tomato (*Solanum lycopersicum*) pollen grains is responsive to heat stress conditions. Plant Reprod 31:367–383. 10.1007/s00497-018-0339-0 29948007 10.1007/s00497-018-0339-0

[CR88] Jiang Y, Lahlali R, Karunakaran C et al (2019) Pollen, ovules, and pollination in pea: Success, failure, and resilience in heat. Plant. Cell Environ 42:354–372. 10.1111/pce.13427 10.1111/pce.13427 30136298

[CR33] Khaitova LC, Mikulkova P, Pecinkova J et al (2024) Heat stress impairs centromere structure and segregation of meiotic chromosomes in *Arabidopsis*. Elife 12:RP90253. 10.7554/elife.90253 38629825 10.7554/eLife.90253 PMC11023694

[CR34] Khan AH, Min L, Ma Y et al (2023) High-temperature stress in crops: male sterility, yield loss and potential remedy approaches. Plant Biotechnol J 21:680–697. 10.1111/pbi.13946 36221230 10.1111/pbi.13946 PMC10037161

[CR35] Kim M, Lee U, Small I et al (2012) Mutations in an *Arabidopsis* mitochondrial transcription termination factor-related protein enhance thermotolerance in the absence of the major molecular chaperone HSP101. Plant Cell 24:3349–3365. 10.1105/tpc.112.101006 22942382 10.1105/tpc.112.101006 PMC3462636

[CR36] Kim J, Park J, Kim H et al (2022) *Arabidopsis* heat shock factor binding protein is required to limit meiotic crossovers and HEI10 transcription. EMBO J 41:EMBJ2021109958. 10.15252/embj.2021109958 10.15252/embj.2021109958 PMC9289711 35670129

[CR37] Koini MA, Alvey L, Allen T et al (2009) High temperature-mediated adaptations in plant architecture require the bHLH transcription factor PIF4. Curr Biol 19:408–413. 10.1016/j.cub.2009.01.046 19249207 10.1016/j.cub.2009.01.046

[CR38] Lei X, Ning Y, Elesawi IE et al (2020) Heat stress interferes with chromosome segregation and cytokinesis during male meiosis in *Arabidopsis thaliana*. Plant Signal Behav 15:1746985. 10.1080/15592324.2020.1746985 32275182 10.1080/15592324.2020.1746985 PMC7238882

[CR39] Lin J-S, Kuo C-C, Yang I-C et al (2018a) MicroRNA160 modulates plant development and heat shock protein gene expression to mediate heat tolerance in *Arabidopsis*. Front Plant Sci 9:68. 10.3389/fpls.2018.00068 29449855 10.3389/fpls.2018.00068 PMC5799662

[CR40] Lin K-F, Tsai M-Y, Lu C-A et al (2018b) The roles of *Arabidopsis* HSFA2, HSFA4a, and HSFA7a in the heat shock response and cytosolic protein response. Bot Stud 59:15. 10.1186/s40529-018-0231-0 29785454 10.1186/s40529-018-0231-0 PMC5962528

[CR41] Lloyd A, Morgan C, Franklin FCH, Bomblies K (2018) Plasticity of meiotic recombination rates in response to temperature in *Arabidopsis*. Genetics 208:1409–1420. 10.1534/genetics.117.300588 29496746 10.1534/genetics.117.300588 PMC5887139

[CR42] Luo J, Jiang J, Sun S, Wang X (2022) Brassinosteroids promote thermotolerance through releasing BIN2-mediated phosphorylation and suppression of HsfA1 transcription factors in *Arabidopsis*. Plant Commun 3:100419. 10.1016/j.xplc.2022.100419 35927943 10.1016/j.xplc.2022.100419 PMC9700127

[CR43] Mácová K, Prabhullachandran U, Štefková M et al (2022) Long-term high-temperature stress impacts on embryo and seed development in *Brassica napus*. Front Plant Sci 13:844292. 10.3389/fpls.2022.844292 35528932 10.3389/fpls.2022.844292 PMC9075611

[CR44] Mathur J, Szabados L, Schaefer S et al (1998) Gene identification with sequenced T-DNA tags generated by transformation of *Arabidopsis* cell suspension. Plant J 13:707–716. 10.1046/j.1365-313x.1998.00059.x 9681013 10.1046/j.1365-313x.1998.00059.x

[CR45] McLoughlin F, Kim M, Marshall RS et al (2019) HSP101 interacts with the proteasome and promotes the clearance of ubiquitylated protein aggregates. Plant Physiol. 10.1104/pp.19.00263 31113833 10.1104/pp.19.00263 PMC6670096

[CR46] Merret R, Carpentier MC, Favory JJ et al (2017) Heat shock protein HSP101 affects the release of ribosomal protein mRNAs for recovery after heat shock. Plant Physiol 174:1216–1225. 10.1104/pp.17.00269 28381501 10.1104/pp.17.00269 PMC5462041

[CR47] Mizoi J, Kanazawa N, Kidokoro S et al (2019) Heat-induced inhibition of phosphorylation of the stress-protective transcription factor DREB2A promotes thermotolerance of *Arabidopsis thaliana*. J Biol Chem 294:902–917. 10.1074/jbc.ra118.002662 30487287 10.1074/jbc.RA118.002662 PMC6341385

[CR48] Morimoto RI (1998) Regulation of the heat shock transcriptional response: cross talk between a family of heat shock factors, molecular chaperones, and negative regulators. Genes Dev 12:3788–3796. 10.1101/gad.12.24.3788 9869631 10.1101/gad.12.24.3788

[CR49] Muthusamy M, Son S, Park SR, Lee SI (2023) Heat shock factor binding protein BrHSBP1 regulates seed and pod development in *Brassica rapa*. Front Plant Sci 14:1232736. 10.3389/fpls.2023.1232736 37719218 10.3389/fpls.2023.1232736 PMC10499616

[CR50] Nguyen TD, Jang S, Soh MS et al (2019) High daytime temperature induces male sterility with developmental defects in male reproductive organs of Arabidopsis. Plant Biotechnol Rep 13:635–643. 10.1007/s11816-019-00559-8

[CR51] Nieto-Sotelo J, Martínez LM, Ponce G et al (2002) Maize HSP101 plays important roles in both induced and basal thermotolerance and primary root growth. Plant Cell 14:1621–1633. 10.1105/tpc.010487 12119379 10.1105/tpc.010487 PMC150711

[CR52] Nover L, Bharti K, Dring P et al (2001) *Arabidopsis* and the heat stress transcription factor world: how many heat stress transcription factors do we need? Cell Stress Chaperones 6:177–189. 10.1379/1466-1268(2001)006<0177:aathst>2.0.co;2 11599559 10.1379/1466-1268(2001)006<0177:aathst>2.0.co;2 PMC434399

[CR53] Ogawa D, Yamaguchi K, Nishiuchi T (2007) High-level overexpression of the *Arabidopsis HsfA2* gene confers not only increased themotolerance but also salt/osmotic stress tolerance and enhanced callus growth. J Exp Bot 58:3373–3383. 10.1093/jxb/erm184 17890230 10.1093/jxb/erm184

[CR54] Prerostova S, Dobrev PI, Kramna B et al (2020) Heat acclimation and inhibition of cytokinin degradation positively affect heat stress tolerance of *Arabidopsis*. Front Plant Sci 11:87. 10.3389/fpls.2020.00087 32133021 10.3389/fpls.2020.00087 PMC7040172

[CR55] Qin F, Yu B, Li W (2021) Heat shock protein 101 (HSP101) promotes flowering under nonstress conditions. Plant Physiol 186:407–419. 10.1093/plphys/kiab052 33561259 10.1093/plphys/kiab052 PMC8154077

[CR56] Queitsch C, Hong SW, Vierling E, Lindquist S (2000) Heat shock protein 101 plays a crucial role in thermotolerance in *Arabidopsis*. Plant Cell 12:479. 10.2307/3871063 10760238 10.1105/tpc.12.4.479 PMC139847

[CR57] Raja MM, Vijayalakshmi G, Naik ML et al (2019) Pollen development and function under heat stress: from effects to responses. Acta Physiol Plant 41:47. 10.1007/s11738-019-2835-8

[CR58] Rana RM, Dong S, Tang H et al (2012) Functional analysis of OsHSBP1 and OsHSBP2 revealed their involvement in the heat shock response in rice (*Oryza sativa* L.). J Exp Bot 63:6003–6016. 10.1093/jxb/ers245 22996677 10.1093/jxb/ers245

[CR59] RECROP (2025) Integrative approaches to enhance reproductive resilience of crops for climate-proof agriculture. Plant Stress 15:100704. 10.1016/j.stress.2024.100704

[CR60] Rieu I, Twell D, Firon N (2017) Pollen development at high temperature: from acclimation to collapse. Plant Physiol. 10.1104/pp.16.01644 28246296 10.1104/pp.16.01644 PMC5373052

[CR61] Rutley N, Poidevin L, Doniger T et al (2021) Characterization of novel pollen-expressed transcripts reveals their potential roles in pollen heat stress response in *Arabidopsis thaliana*. Plant Reprod 34:61–78. 10.1007/s00497-020-00400-1 33459869 10.1007/s00497-020-00400-1 PMC7902599

[CR62] Sage TL, Bagha S, Lundsgaard-Nielsen V et al (2015) The effect of high temperature stress on male and female reproduction in plants. Field Crops Res 182:30–42. 10.1016/j.fcr.2015.06.011

[CR63] Satyal SH, Chen D, Fox SG et al (1998) Negative regulation of the heat shock transcriptional response by HSBP1. Genes Dev 12:1962–1974. 10.1101/gad.12.13.1962 9649501 10.1101/gad.12.13.1962 PMC316975

[CR64] Scharf KD, Berberich T, Ebersberger I, Nover L (2012) The plant heat stress transcription factor (Hsf) family: structure, function and evolution. Biochim Biophys Acta 1819:104–119. 10.1016/j.bbagrm.2011.10.002 22033015 10.1016/j.bbagrm.2011.10.002

[CR65] Shi Y, Mosser DD, Morimoto RI (1998) Molecularchaperones as HSF1-specific transcriptional repressors. Gene Dev 12:654–666. 10.1101/gad.12.5.654 9499401 10.1101/gad.12.5.654 PMC316571

[CR66] Shi W, Yang J, Kumar R et al (2022) Heat stress during gametogenesis irreversibly damages female reproductive organ in rice. Rice (n y) 15:32. 10.1186/s12284-022-00578-0 35763153 10.1186/s12284-022-00578-0 PMC9240181

[CR89] Sidhu BS, Mehrabi Z, Ramankutty N, Kandlikar M (2023) How can machine learning help in understanding the impact of climate change on crop yields? Environ Res Lett 18:024008. 10.1088/1748-9326/acb164

[CR67] Snider JL, Oosterhuis DM, Loka DA, Kawakami EM (2011) High temperature limits in vivo pollen tube growth rates by altering diurnal carbohydrate balance in field-grown *Gossypium hirsutum* pistils. J Plant Physiol 168:1168–1175. 10.1016/j.jplph.2010.12.011 21256621 10.1016/j.jplph.2010.12.011

[CR68] Song C, Lee J, Kim T et al (2018) VOZ1, a transcriptional repressor of DREB2C, mediates heat stress responses in *Arabidopsis*. Planta 247:1439–1448. 10.1007/s00425-018-2879-9 29536220 10.1007/s00425-018-2879-9

[CR69] Storme ND, Geelen D (2020) High temperatures alter cross-over distribution and induce male meiotic restitution in *Arabidopsis thaliana*. Commun Biol 3:187. 10.1038/s42003-020-0897-1 32327690 10.1038/s42003-020-0897-1 PMC7181631

[CR70] Suzuki N, Bassil E, Hamilton JS et al (2016) ABA is required for plant acclimation to a combination of salt and heat stress. PLoS ONE 11:e0147625. 10.1371/journal.pone.0147625 26824246 10.1371/journal.pone.0147625 PMC4733103

[CR71] Tak Y, Lal SS, Gopan S et al (2022) Identification of subfunctionalized aggregate-remodeling J-domain proteins in *Arabidopsis thaliana*. J Exp Bot 74:1705–1722. 10.1093/jxb/erac514 10.1093/jxb/erac514 PMC10010614 36576197

[CR72] Taylor SC, Nadeau K, Abbasi M et al (2019) The ultimate qPCR experiment: producing publication quality, reproducible data the first time. Trends Biotechnol 37:761–774. 10.1016/j.tibtech.2018.12.002 30654913 10.1016/j.tibtech.2018.12.002

[CR73] Teng C, Dong H, Shi L et al (2008) Serine palmitoyltransferase, a key enzyme for de novo synthesis of sphingolipids, is essential for male gametophyte development in *Arabidopsis*. Plant Physiol 146:1322–1332. 10.1104/pp.107.113506 18218968 10.1104/pp.107.113506 PMC2259075

[CR74] Vacca RA, de Pinto MC, Valenti D et al (2004) Production of reactive oxygen species, alteration of cytosolic ascorbate peroxidase, and impairment of mitochondrial metabolism are early events in heat shock-induced programmed cell death in tobacco bright-yellow 2 cells. Plant Physiol 134:1100–1112. 10.1104/pp.103.035956 15020761 10.1104/pp.103.035956 PMC389934

[CR75] Vandesompele J, Preter KD, Pattyn F et al (2002) Accurate normalization of real-time quantitative RT-PCR data by geometric averaging of multiple internal control genes. Genome Biol 3:research0034.1. 10.1186/gb-2002-3-7-research0034 12184808 10.1186/gb-2002-3-7-research0034 PMC126239

[CR76] Wahid A, Gelani S, Ashraf M, Foolad MR (2007) Heat tolerance in plants: an overview. Environ Exp Bot 61:199–223. 10.1016/j.envexpbot.2007.05.011

[CR77] Wang TY, Wu JR, Duong NKT et al (2021) HSP70-4 and farnesylated AtJ3 constitute a specific HSP70/HSP40-based chaperone machinery essential for prolonged heat stress tolerance in *Arabidopsis*. J Plant Physiol 261:153430. 10.1016/j.jplph.2021.153430 33991823 10.1016/j.jplph.2021.153430

[CR78] Wu T, Juan Y, Hsu Y et al (2013) Interplay between heat shock proteins HSP101 and HSA32 prolongs heat acclimation memory posttranscriptionally in *Arabidopsis*. Plant Physiol 161:2075–2084. 10.1104/pp.112.212589 23439916 10.1104/pp.112.212589 PMC3613477

[CR79] Yang D, Li Y, Shi Y et al (2016) Exogenous cytokinins increase grain yield of winter wheat cultivars by improving stay-green characteristics under heat stress. PLoS ONE 11:e0155437. 10.1371/journal.pone.0155437 27203573 10.1371/journal.pone.0155437 PMC4874672

[CR80] Yang F, Fernández-Jiménez N, Tučková M et al (2021) Defects in meiotic chromosome segregation lead to unreduced male gametes in *Arabidopsis* SMC5/6 complex mutants. Plant Cell 33:3104–3119. 10.1093/plcell/koab178 34240187 10.1093/plcell/koab178 PMC8462810

[CR81] Ye Rodriguez, Tran, et al (2000) The developmental transition to flowering represses ascorbate peroxidase activity and induces enzymatic lipid peroxidation in leaf tissue in *Arabidopsis* thaliana. Plant Sci Int J Exp Plant Biol. 158:115–127. 10.1016/s0168-9452(00)00316-2 10.1016/s0168-9452(00)00316-2 10996251

[CR82] Yoshida T, Sakuma Y, Todaka D et al (2008) Functional analysis of an *Arabidopsis* heat-shock transcription factor HsfA3 in the transcriptional cascade downstream of the DREB2A stress-regulatory system. Biochem Biophys Res Commun 368:515–521. 10.1016/j.bbrc.2008.01.134 18261981 10.1016/j.bbrc.2008.01.134

[CR83] Young LW, Wilen RW, Bonham-Smith PC (2004) High temperature stress of *Brassica napus* during flowering reduces micro- and megagametophyte fertility, induces fruit abortion, and disrupts seed production. J Exp Bot 55:485–495. 10.1093/jxb/erh038 14739270 10.1093/jxb/erh038

[CR84] Zhang C, Li G, Chen T et al (2018) Heat stress induces spikelet sterility in rice at anthesis through inhibition of pollen tube elongation interfering with auxin homeostasis in pollinated pistils. Rice 11:14. 10.1186/s12284-018-0206-5 29532187 10.1186/s12284-018-0206-5 PMC5847639

[CR85] Zinn KE, Tunc-Ozdemir M, Harper JF (2010) Temperature stress and plant sexual reproduction: uncovering the weakest links. J Exp Bot 61:1959–1968. 10.1093/jxb/erq053 20351019 10.1093/jxb/erq053 PMC2917059

[CR86] Zupanska AK, LeFrois C, Ferl RJ, Paul A-L (2019) HSFA2 functions in the physiological adaptation of undifferentiated plant cells to spaceflight. Int J Mol Sci 20:390. 10.3390/ijms20020390 30658467 10.3390/ijms20020390 PMC6359015

