# Supplementary material for: Adaptive response to long-term high temperatures during the reproductive development in Arabidopsis thaliana
Source: Planta. 2026 Aug 1;264(3):80. doi: 10.1007/s00425-026-05108-6 (PMC13428784; doi:10.1007/s00425-026-05108-6)
Supplement: Supplementary file 1 — Supplementary file1 (PDF 101 kb) [file 425_2026_5108_MOESM1_ESM.pdf]

Supplementary Table S1. Statistical comparison of the flowering production rate. Negative binomial regression estimated the flowering production rate at each condition, which was compared by ANOVA, followed by the Tukey test for multiple pairwise comparisons.

Comparisons	estimate	S.E.	Df	t-ratio	p-value
CC vs. S1	0.02546	0.00357	76	7.134	< 0.0001
CC vs. S2	0.00592	0.00357	76	1.658	0.3528
CC vs. S3	0.01944	0.00357	76	5.447	< 0.0001
S1 vs. S2	-0.01955	0.00357	76	-5.476	< 0.0001
S1 vs. S3	-0.00602	0.00357	76	-1.687	0.3375
S2 vs. S3	0.01352	0.00357	76	3.789	0.0017

Supplementary Table S2. Statistical comparison of the primary branch growth rate. Exponential growth model estimated the growth rate for plant, which was compared by ANOVA, followed by the Tukey test for multiple pairwise comparisons.

Comparisons	estimate	S.E.	Df	t-ratio	p-value
CC vs. S1	0.1764	0.0204	76	8.655	< 0.0001
CC vs. S2	0.2247	0.0204	76	11.024	< 0.0001
CC vs. S3	0.2425	0.0204	76	11.896	< 0.0001
S1 vs. S2	0.0483	0.0204	76	2.369	0.0919
S1 vs. S3	0.0660	0.0204	76	3.240	0.0094
S2 vs. S3	0.0178	0.0204	76	0.872	0.8195

Supplementary Table S3. List of primers used for RT-qPCR and genotyping

HsfA1d RT-F	GGATTCAACACCAGTGGACAATG
HsfA1d RT-R	AGGAGACCCATCTGTTGAGTCAG
HSFA2 qPCR F	TGAAGGGTTTTTAGCAGGACAA
HSFA2 qPCR R	CTGCAAACCCATGTTCCTCC
HSFA4c qPCR F NEW	CAAGCTCACTTCCACCTTTCCT
HSFA4c qPCR R NEW	TGTTGTTTTCGCTCCAAGCA
HSBP Forward	TGATTCTGCCTCCCATGTCA
HSBP Reverse	GCTGATATGACTGCTTTTGTCCA
PP2a_A3 qPCR F	AAGCGGTTGTGGAGAACATGATACG
PP2A_A3 qPCR R	TGGAGAGCTTGATTTGCGAAATACCG
TMA7 qPCR F	AGCTAAGTCAGTCTCGTCGT
TMA7 qPCR R	AGCAAGGAGGAAAGGCGAAA
APX2 qPCR F	ACTCCTTGTCAGCAAACCCGAG
APX2 qPCR R	CTTGATGATCCTCTCTTTCTCCCA
HSP101 qPCR F	ATGACCCGGTGTATGGTGCTAG
HSP101 qPCR R	CGCCTGCATCTATGTAAACAGTG
TAA1 new qpcr F	TGGCTAGGGACGAAGGAAGA
TAA1 new qpcr R	GCTGACTCGGACATGCTTCT
HSP70 qpcr F	CCGTCTTCGATGCTAAGCGTCT

HSP70 qpcr R	AACCACAATCATAGGCTTCTCACC
hot1-3-F	CATGCCTCCTCGCTCTCTCGCAATTC
hot1-3-R	TTTGGCCAAGTCTCGTCAC
KM2	CAGTCATAGCCGAATAGCCTCTCCACCC
SALK_066374-LP/ SALK_099583-LP	AATAATGCGGCAAAAGAGGAG
SALK_066374-RP/ SALK_099583-RP	CTGCTTGCTCAACCAAATCTC
LBb1.3	ATTTTGCCGATTTCGGAAC
